# The Utility of Electrochemical Systems in Microbial Degradation of Polycyclic Aromatic Hydrocarbons: Discourse, Diversity and Design

**DOI:** 10.3389/fmicb.2020.557400

**Published:** 2020-10-23

**Authors:** Da-Cheng Hao, Xiao-Jing Li, Pei-Gen Xiao, Lian-Feng Wang

**Affiliations:** ^1^School of Environmental and Chemical Engineering, Dalian Jiaotong University, Dalian, China; ^2^Agro-Environmental Protection Institute, Ministry of Agriculture and Rural Affairs/Key Laboratory of Original Agro-Environmental Pollution Prevention and Control, MARA/Tianjin Key Laboratory of Agro-Environment and Agro-Product Safety, Tianjin, China; ^3^Institute of Medicinal Plant Development, Chinese Academy of Medical Sciences, Beijing, China

**Keywords:** polycyclic aromatic hydrocarbon, microbial fuel cell, anode modification, electroactive microbe, degradation

## Abstract

Polycyclic aromatic hydrocarbons (PAHs), especially high molecular weight PAHs, are carcinogenic and mutagenic organic compounds that are difficult to degrade. Microbial remediation is a popular method for the PAH removal in diverse environments and yet it is limited by the lack of electron acceptors. An emerging solution is to use the microbial electrochemical system, in which the solid anode is used as an inexhaustible electron acceptor and the microbial activity is stimulated by biocurrent *in situ* to ensure the PAH removal and avoid the defects of bioremediation. Based on the extensive investigation of recent literatures, this paper summarizes and comments on the research progress of PAH removal by the microbial electrochemical system of diversified design, enhanced measures and functional microorganisms. First, the bioelectrochemical degradation of PAHs is reviewed in separate and mixed PAH degradation, and the removal performance of PAHs in different system configurations is compared with the anode modification, the enhancement of substrate and electron transfer, the addition of chemical reagents, and the combination with phytoremediation. Second, the key functional microbiota including PAH degrading microbes and exoelectrogens are overviewed as well as the reduced microbes without competitive advantage. Finally, the typical representations of electrochemical activity especially the internal resistance, power density and current density of systems and influence factors are reviewed with the correlation analysis between PAH removal and energy generation. Presently, most studies focused on the anode modification in the bioelectrochemical degradation of PAHs and actually more attentions need to be paid to enhance the mass transfer and thus larger remediation radius, and other smart designs are also proposed, especially that the combined use of phytoremediation could be an eco-friendly and sustainable approach. Additionally, exoelectrogens and PAH degraders are partially overlapping, but the exact functional mechanisms of interaction network are still elusive, which could be revealed with the aid of advanced bioinformatics technology. In order to optimize the efficacy of functional community, more advanced techniques such as omics technology, photoelectrocatalysis and nanotechnology should be considered in the future research to improve the energy generation and PAH biodegradation rate simultaneously.

## Introduction

Polycyclic aromatic hydrocarbons (PAHs), especially high molecular weight (HMW) PAHs, are carcinogenic and mutagenic organic compounds that are difficult to degrade (Liang et al., 2020; Sharma et al., 2020). The physical and chemical remediations (e.g., gas stripping method, leaching method and chemical agent addition) are cost-intensive and environmentally non-friendly. Microbial remediation is a common method for the treatment of PAHs in soil, sediment, water and wastewater. However, microbial remediation technology has many disadvantages, e.g., low abundance, poor diversity, poor activity, slow growth of PAH degrading microbe, and poor bioavailability of PAH in soil and water (Hao et al., 2016, 2018a). The cost of adding nutrients, electron acceptors or co-substrates to enhance the activity of local microorganisms is unbearable, and the additives tend to diffuse away from the target PAHs. The phytoremediation of PAH also has no ideal effect and cost effectiveness (Liu et al., 2019; Zhao et al., 2019). At present, the promising solution is to use bioelectrochemical system (BES), in which microbial community participates in the detoxification/valorization of organic contaminants, and microbes exchange electrons with the electrode (Zhou et al., 2020). If the type of BES is microbial fuel cell (MFC), the electric current is generated via microbial oxidation of organic substrates at the anode. If the type of BES is microbial electrolysis cell (MEC), when electric energy is provided, hydrogen is produced at the cathode due to electrons generated by microbial oxidation of organic substrates at the anode. So far, for various settings MFC is the predominant BES used in degrading PAH (Li et al., 2019a; Zhou et al., 2020), and there are few reports concerning about the use of MEC and other BESs for this purpose, thus this article focuses on MFC.

The use of MFCs in the PAH degradation of soil, groundwater and sediment is booming. The main advantages of MFCs are as follows: First, the terminal electron acceptor (TEA) is the inexhaustible solid electrode, and the bioelectricity is continuously produced (Liu et al., 2019; Yu et al., 2019), thus the self-support apparatus can be developed and used in the remote area for PAH remediation. Second, indigenous microbes of bioremediation sites act as electrogens/electrotrophs and degraders, and their growth and activity can be induced *in situ* (Kirmizakis et al., 2019). These autochthonous strains are more adapted to the local niche, which, unlike exotic bioaugmentation strains, will not be removed soon. Third, in soil/sediment MFCs, the actual mechanism of electroremediation is that the PAH oxidation and biodegradation processes are driven by the directed transfer of electrons (Zhao et al., 2018; Xu et al., 2019). In this article, how MFCs of different configurations cope with PAHs is firstly emphasized, since how to improve the degradation rate of PAH is our most concern; then the microbial diversity in MFCs is summarized, including both PAH degrading microbe and exoelectrogen, which is followed by the review of electrochemical property of various MFCs, with the hope of increasing power generation. The anode modification/improvement is the current research focus, while the biocathode is less studied. Lastly the future research in BES and PAH bioremediation is prospected.

## PAH Degradation

### Separate Degradation vs. Mixed Degradation

**FIGURE 1:**
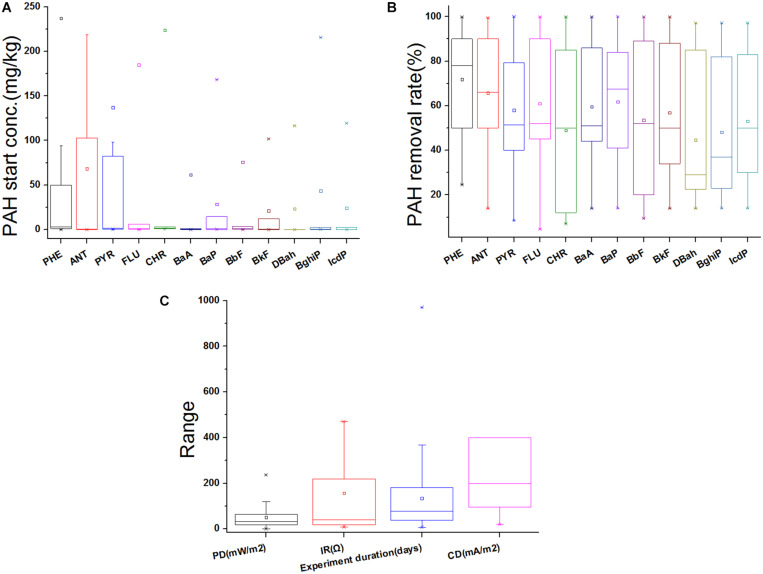
Summary of PAH removal in MFCs. **(A)** Start concentration of each PAH; **(B)** Removal rate (%) of each PAH after MFC treatment; **(C)** power density (PD), current density (CD), internal resistance (IR), and experiment duration reported in recent MFC studies. Boxes represent 25–75% of the data, middle lines the median value, squares the average, and ends represent the minimal and maximal values (1.5 times less or more than the lower or upper quantiles). Outlier is shown in multiplication sign. Three rings PAH: PHE, phenanthrene; ANT, anthracene; Four rings: PYR, pyrene; FLU, fluoranthene; CHR, chrysene; BaA, benzo(a)anthracene; Five rings: BaP, benzo(a)pyrene; BbF, benzo(b)fluoranthene; BkF, benzo(k)fluoranthene; DBah, dibenzo(a,h)anthracene; Six rings: BghiP, benzo(g,h,i)perylene; IcdP, indeno(1,2,3-cd)pyrene.

Unlike the open circuit anaerobic system, the electro-transformation in BES can dramatically enhance the degradation of obstinate PAHs (Daghio et al., 2017). For the single PAH, the laboratory scale MFC is especially useful in the detailed study of bioelectrochemical degradation and influence of single factor. For example, in a 100 mL single-chamber MFC, the HMW PAHs, e.g., pyrene (PYR), were mineralized slower than LMW PAHs [e.g., phenanthrene (PHE)], as microbial communities prefer the latter as the sole carbon source (Zhou et al., 2020) (Supplementary Table 1). At 120 h of MFC operation, the maximal removal of naphthalene (NAP, 97.6%) and PHE (42.9%) was much higher than that of PYR (22%). In recent MFC studies, despite higher start concentration of LMW PAHs (e.g., Li et al., 2018, 2019a; Liang et al., 2020) (Figure 1A), three-ring PAHs were more easily degraded than most examined HMW PAHs (e.g., Hamdan et al., 2017; Liu et al., 2019; Yu et al., 2019) (Figure 1B). In fact, the enhancement efficiency of PAH degradation is mainly determined by the hydrophilicity, toxicity and bioavailability.

Various PAHs, including low molecular weight (LMW) and high (H) MW ones, usually mix in polluted sediments and soils. The well aged and easily assimilative carbon sources are commonly used to co-metabolize mixed PAHs. In a multi-anode soil MFC, glucose was added to increase the bioelectrochemical degradation of 16 priority control PAHs, especially for fluorine (FLN) and fluoranthene (FLU) (Li et al., 2016a). Additionally, a recent study found that even LMW PAH could be as the co-metabolic substrate to promote the degradation of HMW ones. In the mixed degradation, the removal rate of PHE was decided by the ratio of NAP vs. PHE or PYR (Zhou et al., 2020). The highest degradation 89.2% of PHE was attained at 120 h with NAP-PHE 1:2. while the ratio of NAP-PYR 1:4 led to the highest degradation 51.4% of PYR. The degradation of PHE and PYR was also reciprocally promoted to a lesser extent. When LMW PAHs are used by anaerobes as co-metabolic substrates, more degradation enzymes can be generated (Gao et al., 2019). In MFC, the production of methylation related enzymes could be enhanced by the methylation degradation of NAP in the NAP-PHE mixture, and methylation could take the lead in PHE degradation. PHE was transformed to carbon dioxide, and 4-propylphenol, p-cresol, and phenol were intermediates (Zhou et al., 2020). PYR underwent hydroxylation and methylation with intermediates such as 4,5-dihydropyrene, 4,5-dimethylphenanthrene, and 4-methylphenanthrene (Liang et al., 2014; Zhou et al., 2020). PHE was also an intermediate product of PYR degradation in MFC. In a word, the superiority of mixed degradation is to directionally induce the special enzymes that accelerate the PAH degradation.

### The Effect of Anode Modification

**FIGURE 2:**
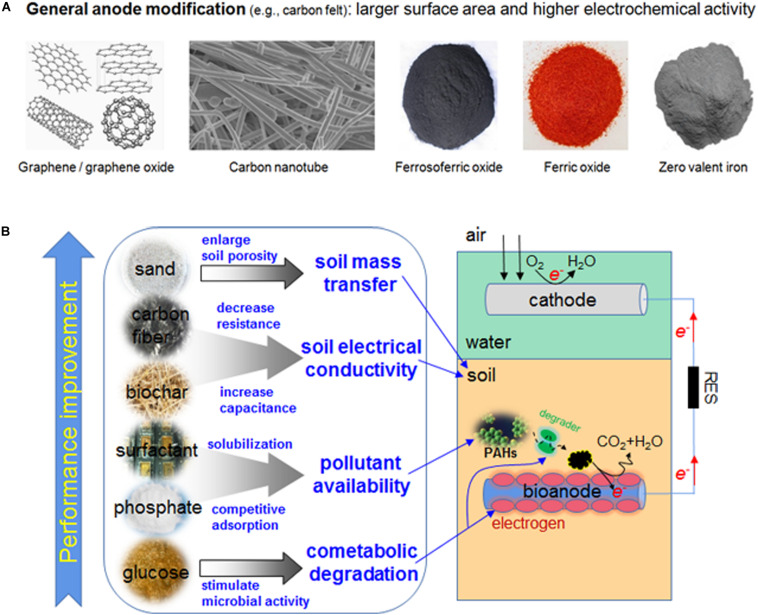
**(A)** Examples of materials used for anode modifications of MFC; **(B)** example methods of improving MFC performance in PAH degradation.

The PAH degradation could be improved via anode modification. Graphite felt (GF) is commonly used in electrode design, but there could be better choices. The carbon nanomaterials, e.g., graphene (GR), graphene oxide (GO, used after reducing it to reduced graphene oxide), and carbon nanotube (CNT) (Figures 2A,B), provide larger specific surface area for microbial growth and biofilm formation, and their conductivity and electrochemical capacity are also outstanding. In a sediment MFC (SMFC) study, when GR, GO, and CNT were used as anode material, the PHE/PYR removal near anode was higher than that of GF-SMFC (Liang et al., 2020). In the sediments less than 1 cm from the anode, the PYR removal of GF-SMFC (42.3%) was much lower than that of GO-SMFC (69.6%), GR-SMFC (68.2%), and CNT-SMFC (66.7%). If sediments were far away from the anode, due to the difficulty in mass transfer, the PHE/PYR removal declined. Granular activated carbon (GAC), a porous sorption material, can be used to produce electrodes with large surface area and enhanced capability of sequestering and degrading pollutants (Mohan et al., 2014). In a MFC with GAC anode, the removal of C12-C16 (99.49%, including three- and four-ring PAHs), C16-C21 (99.86%, four- and five-ring PAHs) and C21-C35 (97.08%, PAHs with five and six rings) was much more than that with non-GAC anode (Kirmizakis et al., 2019). The removal of heaviest aromatic fraction (C35–C44), corresponding to 0.56% of total aromatic hydrocarbon, was lower (54.17%).

Iron-containing cytochrome C and Fe–S protein are the essential components of electron transport chain in most exoelectrogens (Li and Gao, 2010). The MFC anode can be modified by iron oxides (Fe_3_O_4_/Fe_2_O_3_) and zero valent iron (ZVI); the kinetic activity of anode reaction can be improved by iron oxides, and a strong anaerobic environment is established by ZVI to facilitate the electron transfer. The microbial growth and metabolism were promoted by electrons from ZVI (Yu et al., 2019). Bentonite, a cost effective sorption material, was used as a supporting carrier of ZVI, on which the dispersion and stability of ZVI particles are better, and the synergistic adsorption of composites can be brought into full play (Kumar et al., 2012). In a soil MFC, The removal rate of anthracene (ANT), PHE and PYR was 31.56%, 28.53%, and 23.28% respectively in GF + Fe3O4 (GFF) group; in GF + bentonite-Fe (GFB) group, the corresponding removal rate was increased to 36.62, 32.48, and 26.24%, respectively (Yu et al., 2019). These values were higher than those of GF group, suggesting the effect of anode modification.

As for anode modification, most studies focus on the physical and chemical properties of the material *per se*, e.g., superficial area and conductivity, etc.; by improving them, more reactive sites could be provided and the electron transfer could be accelerated. Actually, the biocompatibility of anode material is equally important for the improvement of anodic performance, as the role of anode in the bioelectrochemical degradation is also determined by the activity of special microorganisms on the surface of anode.

### The Effect of MFC Configuration

The optimization of MFC design is conducive to giving full play to the degradation competence of microbes. For example, the multi-anode configuration could increase PYR/PHE removal rate (Li et al., 2014; Xu et al., 2019); when compared to natural attenuation, the larger anode surface area and boosted charge accumulation in multi-anode configuration enhanced the degradation of total petroleum hydrocarbon (TPH), PAH, and *n*-alkanes (Li et al., 2014). The area of cathode can afford larger projected area of anode in terms of water medium MFC systems.

The limited mass transfer in sediment or soil is a major challenge of MFC (Zhao et al., 2017), especially when the natural water/groundwater flow rate is low. In MFC the metabolic substrate around the anode is continuously consumed, but very often the distant pollutants can only be transferred at a very slow rate, thus the oxidation reactions and electroactive bacteria (EAB) activities cannot be sustained on the anode. The poor mass transfer also led to the accumulation of protons in the sediment near the anode (Hsu et al., 2013), and the power generation and PAH degradation are negatively affected. In fact, the soil MFC remediation of petroleum hydrocarbons is only effective within 1 cm from anode (Wang et al., 2012). In another soil MFC, after 64 d the removal rate of petroleum hydrocarbons was 73.1–78.3% within 1 cm from anode and decreased to 51.5–56.8% at 5 cm from anode (Lu et al., 2014). Some studies were performed to improve substrate mass transfer in soil/sediment MFCs. With multi-anode arrangement, the performance of scaled-up or field-scale MFC was improved, and both remediation radius and electric charges were increased (Hsu et al., 2013; Zhao et al., 2017). However, the substrate mass transfer was still poor. In another MFC study, the soil porosity was increased and the ohmic resistance declined by mixing soil and sand at a mass ratio 2:1 (Li et al., 2015) (Figure 2B); after 135 days the degradation rate of PAHs increased by 2.7 times. Of note is the removal rate of recalcitrant five ring PAH dibenzo(a,h)anthracene (DBah, C_22_H_14_) and six ring benzo(g,h,i)perylene (BghiP, C_22_H_12_) was as high as 22.5 ± 1.5%. Mixing soil and sand at a mass ratio 5:1 failed to improve the MFC performance. Moreover, mixing soil and sand diluted the easily degraded organic matter near anode, and the MFC performance could be adversely affected. Thus, the balance between enhanced mass transfer and substrate availability need to be overall considered in the application of bioelectrochemical remediation.

Alternatively, the contaminated soil and conductive carbon fiber were mixed to increase the degradation of 16 priority PAHs (Li et al., 2016c), as the electron transfer from the soil to anode and power output were improved (Figure 2B), and the effective remediation radius was enlarged from 6 to 20 cm; the air-cathode area was not changed. In order to enhance the mass transfer in the topsoil, a separate aqueous layer in sediment around the anode can be created by a fabric baffle (Lee et al., 2015), which outperformed embedding MFC anode in the sediment and increased the maximal power by 6.6 times. This approach could be applied in the *ex situ* remediation of polluted soil, since it is difficult to construct the water layer in the large-scale polluted site. A plant-driven MFC (New-PSMES), containing a stand-alone anode column and *Iris pseudacorus* in PAH-polluted sediment, is a new attempt (Liu et al., 2019). In traditional plant MFCs, the organic matter of rhizosphere is useful carbon source for co-metabolism of intransigent pollutants and electricity generation (Yan et al., 2015; Adelaja et al., 2017). In this novel attempt, the anode is directly put in the rhizosphere. Can the plants improve the substrate mass transfer? Theoretically, it is possible, as water can be drawn through the sediment via plant transpiration, and substances can be moved with water. In the sand-filled anode column of New-PSMES, water is lost via plant transpiration, and the water-permeable GF at the column bottom permits outside pore water in sediments to be sucked into the column. There is hydraulic pressure between two sides of anode, as the water permeability of outside sediment is lower than that of sand within column, thus the pore water is moved upward by the above two factors, along with fine particles and adsorbed organic pollutants. PAHs are less water soluble, but the adsorbed PAH can be moved to the anode region with fine particles and water. The addition of anode column resulted in better substrate mass transfer and a larger remediation range of New-PSMES. The removal rates of PHE (62.98%) and PYR (57.02%) were significantly higher than those of plant-MFC without column (PHE 47.7%, PYR 43.1%) and MFC without plant/column (PHE 42.9%, PYR 41.8%). In New-PSMES, PAHs far from anode are bio-degraded *in situ*, and PAHs near anode are mainly degraded via electrochemical reactions (Liu et al., 2019). The role of plant absorption/adsorption in PAH removal is negligible (<0.1%). The overall microbial activity in New-PSMES was increased by the “plant pump” via the improved mass transfer.

Biochar is a kind of charcoal used as soil modifier, which can help plants and microorganisms grow. The biochars prepared from chicken manure (CB), wheat straw (SB) and wood sawdust (WB) were supplemented in soil MFCs at 2% mass ratio (Li et al., 2019a). CB is rich in the mineral nutrition and phosphorus, SB is rich in nitrogen and of the highest molecular polarity, while WB has the largest surface area, and the most charges can be obtained. Compared to the control, CB, SB and WB increased the removal of four dominant PAHs (PHE, FLU, PYR and CHR) by 49–79%, 32–57%, and 26–44%, respectively. The degradation of total PAHs increased by 67, 38, and 36% in CB, SB and WB respectively as compared to control. Similar to the role of carbon fiber, the biochar actually constructs a conductivity network to accelerate electron transfer (Figure 2B) and thus increase the validation of biocurrent stimulation in addition to above-mentioned physicochemical properties. Biochar can also promote direct interspecies electron transfer (DIET) (Wang et al., 2020). On the one hand, DIET by biochar increases the current density of system, which provides a stronger stimulation on the activity of degrading bacteria. On the other hand, the enhanced DIET could pull the equilibrium of degradation reaction to accelerate oxidation of PAHs.

Different from the water medium bioelectrochemical system, the pollutant removal and electricity generation of soil or sediment systems suffer from great mass transfer obstacle, although very satisfactory performance is gained on the surface of electrode. Therefore, the enlargement of effective remediation radius is the first to bear the brunt if we want to apply bioelectrochemical technology in soil and sediment.

### Combined Use of Phytoremediation and MFC

**FIGURE 3:**
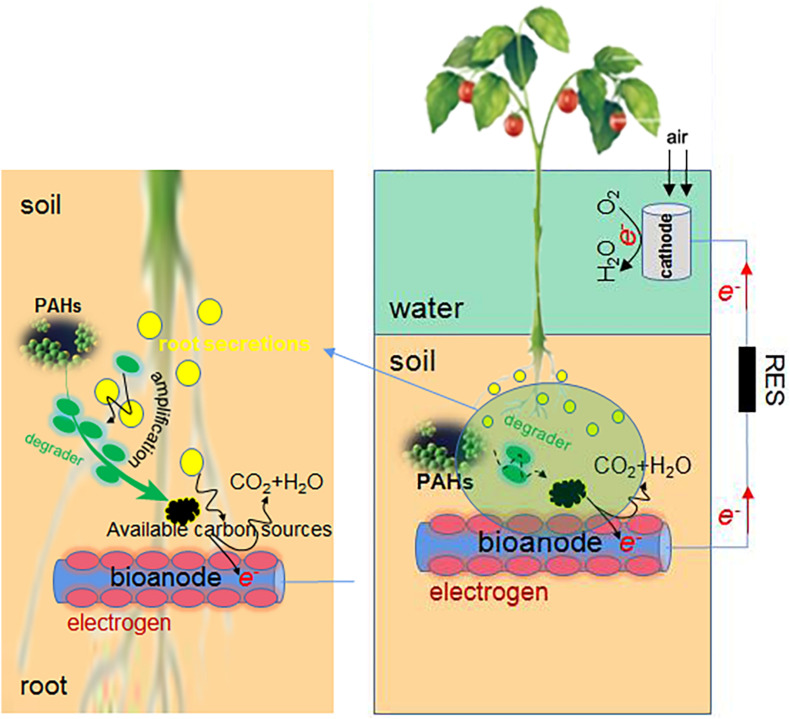
The combined use of phytoremediation and MFC in PAH elimination.

The PAH degradation efficiency of electrochemical remediation is usually higher than that of microbial remediation, and phytoremediation is least efficient. These methods can be combined flexibly (Figure 3). For example, the combined use of MFC and hygrophyte *Aglaonema commutatum cv. silver queen* dramatically improved the degradation of PHE/PYR in the crude oil polluted soil (Zhao et al., 2019). When the macrophyte *Vallisneria spiralis* is used in SMFC, PAH concentrations of plants were much lower than those of sediments (Xu et al., 2019), partly due to their high *K*_ow_ (octanol-water partition coefficient) (Ramdine et al., 2012). The plant uptake of PAHs is not worth mentioning. The advantages of MFC plus *V. spiralis* are as follows. Firstly, the desorption of PAHs from sediment particles is facilitated by the LMW organic acids of rhizosphere, and the growth of PAH degrading microbes is also promoted by these root exudates since they function as co-metabolic substrates (Zhang and Lo, 2015). The desorbed PAHs are more easily attached and degraded by MFC microbes. Next, the radial oxygen release (ROL) from *V. spiralis* triggers the sulfur oxidation and significantly increases sulfate in sediments, which is followed by the sulfate reduction via sulfate-reducing bacteria (SRB) such as *Desulfobulbus*, *Desulfovibrio*, and *Desulfobacca* (Supplementary Table 2). The PAH degradation can be promoted by the enhanced sulfate reduction. Thirdly, the pore water Fe^2+^ is increased and Fe^3+^ is reduced in sediments by macrophyte treatment, as iron is driven by ROL from sediments to pore water. The desorbed Fe^3+^ accumulates in the rhizosphere as electron acceptor, and the PAH degradation around rhizosphere is favored. Additionally, the rhizosphere environment is conducive to the enrichment of aerobic PAH degraders, e.g., *Flavobacteria*, *Agrobacterium* (Zhao et al., 2016), *Vogesella* (Yan et al., 2015), and *Bradyrhizobium*. Lastly, the methanogenic microbes are reduced by macrophyte treatment, and the improved redox potential of sediments inhibits the methane generation; with reduced competition of carbon source, the PAH degradation is promoted. In fact, only single application of bioelectrochemical remediation technology faces more challenges, yet the synthesis technology that combines MFC with present successful techniques would have more opportunities.

### The Effect of Chemical Reagents

Besides the electrical stimulation of MFC, the stimulation of some chemical reagents, either natural or synthetic ones, could further enhance the bio-electroremediation efficiency. In a cylindric SMFC, methanol added to the sediment at the bottom of MFC reactors (Zhao et al., 2018) increased the removal of PHE, PYR and chrysene (CHR, C_18_H_12_) to 87, 70, and 65% respectively. As an LMW co-substrate and carbon source, methanol was used by SRB and methanogen within the sediment, and the PAH dissipation is promoted due to the co-metabolic biodegradation and reduced competition of carbon source. The phthalic acid di(2-propylpentyl) ester, an intermediate of PAH degradation, was identified in the sediment, which needs to be concerned in the process of PAH removal.

The proper use of surfactant could increase the partition of PAHs into the aqueous phase. In a single-chamber plant-MFC, the highest removal efficiencies of PHE and PYR (54.2 and 48.4%, respectively) at 5 mmol/kg surfactant β-cyclodextrin were higher than those (45.5 and 41.4%) at 5 mmol/kg Tween 80 (Zhao et al., 2019). The β-cyclodextrin was also superior to glyceryl monostearate and cetyltrimethylammonium bromide (CTAB) during MFC remediation of soil TPHs (Li et al., 2018). At concentrations below the critical micelle concentration, the bioremediation of soil PAH was not significantly improved by Tween 80 (Cheng et al., 2018). The efficient micelles (hydrophobic interior vs. hydrophilic exterior) cannot be formed in the water due to the soil adsorption of part of Tween 80, thus the PAH solubility cannot be increased. Conversely, organic compounds can be included in the truncated cone structure of β-cyclodextrin at the ratio of around 1:1, and the hydrophobic interior and hydrophilic exterior ensure the solubilizing effect (Ni et al., 2014). Only one PAH molecule can be accommodated in the β-cyclodextrin cavity, therefore the maximal PAH removal could be attained by increasing the concentration of β-cyclodextrin. The addition of β-cyclodextrin was better than the glucose co-metabolism in enhancing oil removal from soil (Zhao et al., 2019). Certainly, the cost and environmental friendliness of surfactant also need to be considered in large scale MFC remediation.

In an MFC study of aged soil, five types of surfactants showed differential effects in increasing the PAH bioavailability (Li et al., 2018). The efficacy of ampholytic surfactant lecithos was the best in promoting the degradation of 16 priority PAHs, including fluoranthene (FLU, C_16_H_10_), PYR, and CHR of four aromatic rings, as well as benzo(b)fluoranthene (BbF) and benzo(k)fluoranthene (BkF) of five rings. After surfactant treatment, the total removal of 16 PAHs increased to 90%, but CTAB (cationic surfactant) and glyceryl monostearate (nonionic) were not efficient. However, the enhancement efficiency of different surfactants on the bioelectrochemical degradation of PAHs need to be further addressed, since this study only selected one concentration for five kinds of surfactant.

## Microbial Diversity in MFC

### Anode Microbiota Involved in PAH Degradation

It is not surprising to find some changes of microbial community composition and structure in MFC experiments. Any initial community is subjected to the influence of selection, drift, dispersal, and mutation, etc. Microbes important for the biofilm construction, electrochemical activity, and PAH removal could increase in abundance, while others without essential roles could decline in proportion. The anaerobic sludge from the petrochemical wastewater treatment plant, mixed with glucose medium, can be used as the MFC inoculum to form an anode biofilm (Zhou et al., 2020). Anaerolineaceae (phylum: Chloroflexi), *Clostridium* (phylum: Firmicutes), *Roseiflexus* (Chloroflexi), Cyanobacteria, *Methylophilus* (class: Betaproteobacteria), and Chitinophagaceae (phylum: Bacteroidetes) were dominant in anode community (Supplementary Table 2), most of which are involved in PAH degradation (van der Waals et al., 2017; Blanco-Enriquez et al., 2018). In a SMFC study, the protein concentrations on the anodes modified with carbon nanomaterials (GR, GO and CNT) were much higher than that on the unmodified anode (Liang et al., 2020), and a special microbial community was selected and enriched on the anode. Among 3391 shared OTUs and at the level of phylum, Proteobacteria predominated (29.05% abundance) over Chloroflexi (13.89%), Acidobacteria (6.4%), and Actinobacteria (5.96%), and the abundance of Bacteroidetes (4.66%), Planctomycetes (4.48%) and Firmicutes (4.10%) were also increased. The abundance of PAH degrading *Thauera* and *Diaphorobacter* (Betaproteobacteria), SEEP-SRB1, *Tumebacillus* (Firmicutes), *Lysobacter*, *Acinetobacter* and *Pseudomonas* (Gammaproteobacteria), and *Desulfobulbus* (Deltaproteobacteria) was increased on anodes, some of which are also involved in electron transfer (Kleindienst et al., 2012; Zhao et al., 2018; Rabodonirina et al., 2019).

In a 60 mL dual-chamber MFC with proton exchange membrane, *Pseudomonas* was dominant in both planktonic and biofilm parts (Sharma et al., 2020). PHE was quickly adsorbed and assimilated by surfactant producing *Pseudomonas* (Rabodonirina et al., 2019). In a bacterial consortium, *Pseudomonas* and *Rhodococcus* (Actinobacteria) synergistically degrade PAHs partially due to their bioemulsifying properties (Isaac et al., 2015). Proteobacteria and Firmicutes species are commonly found in PAH polluted sites, where hydrogenotrophic or acetotrophic microbes co-inhabit and synergistically participate in the PAH degradation (Toth and Gieg, 2018; Sharma et al., 2020). When three types of biochar were added in the MFC, Actinobacteria genera, e.g., *Actinotalea*, *Georgenia*, *Mycobacterium*, *Nitriliruptor*, and *Rhodococcus*, were found to take part in the anaerobic PAH degradation (Li et al., 2019a). The unidentified Anaerolineaceae genus also participated in the PAH degradation upon biochar treatment.

The abundance of *Rhizobium* (Alphaproteobacteria) and *Thauera*, the endosymbiotic nitrogen-fixer, increased in the bacterial community of anode biofilm (Sharma et al., 2020). These root associated microbes elicited the mRNA expression of hydrocarbon degrading enzymes such as alkane monooxygenase and naphthalene dioxygenase, and produced organic acids, siderophore, and phytochelatin to facilitate PAH dissipation (Bisht et al., 2015; Thomas and Cébron, 2016). As above-mentioned, BES could be combined with phytoremediation to trigger the PAH removal (Xu et al., 2019).

In addition, *Dechloromonas* species (Betaproteobacteria) anaerobically degrade aromatic compounds (Yan et al., 2017), participate in electron transfer and abundantly inhabit on the anode of SMFC (Xu et al., 2019). Many other PYR degrading anaerobes were enriched in SMFC treated sediments, e.g., *Mycobacterium* (Actinobacteria) and *Nevskia* (Gammaproteobacteria) (Yan et al., 2017; Wang et al., 2018). In wastewater, *Hyphomicrobium* and *Rhodoplanes* (Alphaproteobacteria) directly take part in the PHE degradation via indigenous bioaugmentation (Li et al., 2018). These genera were enriched during SMFC operation (Xu et al., 2019). The addition of methanol increased the abundance of PAH degraders unclassified Peptostreptococcaceae and *Clostridium* (Firmicutes) simultaneously in the anode biofilm (Zhao et al., 2018).

### Anode Microbe as Exoelectrogen

The biofilm microbes on the anode surface may have electrical activity and participate in electron transfer, thus forming the electroactive biofilm. Many Proteobacteria and Firmicutes species, rather than Actinobacteria and Acidobacteria, are electrogenic (Yu et al., 2017). *Pseudomonas* showed electrical activity in MFC and was a key initiator of biofilm formation (Morris et al., 2009; Venkidusamy et al., 2016; Rabodonirina et al., 2019). *Pseudomonas* produces phenazine (stimulated by graphene) and pyocyanin, which, along with vigorous biofilm as the electronic mediator, participate in the extracellular electron transfer (EET) (Adelaja et al., 2014). Other Gammaproteobacteria, e.g., *Pseudoxanthomonas* and *Thermomonas*, may also be involved in the power output of soil MFC (Yu et al., 2019).

xIn a plant-SMFC, the closed circuit condition increased the abundance of Bacilli and *Clostridia* (Firmicutes) (Xu et al., 2019), implying the presence of an ecological niche for the growth and function of exoelectrogen (Ishii et al., 2017). Geobacteraceae species have long been known as exoelectrogen under closed circuit (Zhao et al., 2018; Xu et al., 2019; Yu et al., 2019). LMW organic acids can be the electron donor of *Geobacter*, and the electric current was generated via direct/indirect electron transfer to anode (Ding et al., 2016). There are syntrophic interactions between *Geobacter* and *Clostridium*, and the cellulose fermentation leads to the power generation (Kouzuma et al., 2013). The robust growth of electrogenic bacteria around the anode makes it a sink of electrons to accelerate the degradation of organic pollutants. Other exoelectrogens (Supplementary Table 2), e.g., *Kocuria* (Actinobacteria; Luo et al., 2015), *Bacteroides* (Toczylowska-Maminska et al., 2018), *Proteiniphilum* (Bacteroidetes; Yu et al., 2019), *Geobacillus* (Firmicutes; Venkidusamy et al., 2016), *Shewanella* (Gammaproteobacteria), *Rhodopseudomonas* (Alphaproteobacteria), *Geoalkalibacter* (Deltaproteobacteria; Hamdan et al., 2017), *Ochrobactrum* and *Azospirillum* (Alphaproteobacteria) (Zhou et al., 2016; Li et al., 2019a), and *Escherichia* sp. (Gammaproteobacteria; Li et al., 2014), also have higher abundance under power output condition.

In MFCs, the abundance of SRB *Desulfatitalea* (Li et al., 2019a), *Desulfobulbus* and *Desulfovibrio* (Liu et al., 2019; Xu et al., 2019) increased under closed circuit. These electrogenic bacteria transfer electrons to the anode via sulfur cycling. The PAH degradation is also promoted by sulfur cycling, when the anode is electron acceptor and PAHs are electron donor. *Desulfobulbus* and *Desulfovibrio* could be involved in PAH degradation (Zhao et al., 2018; Liu et al., 2019). In SMFCs, the abundance of *Gallionella* (Betaproteobacteria) significantly increased on the anode, which is implicated in Fe oxidation (Fleming et al., 2018; Xu et al., 2019). Fe cycling bridges organic pollutant degradation, anode and Fe in MFC (Kato et al., 2010). Fe^3+^/Fe cycling, via indirect electron transfer, is involved in power output and PAH degradation started by the increased abundance of *Geobacter* (Zhou et al., 2016). Fe^3+^ and sulfate assist in PAH removal in SMFC, and the anode is main TEA triggering PAH degradation and maintaining stable voltage (Hamdan et al., 2017).

In an MFC for treating groundwater PAHs, the abundance of Fe^3+^ reducers, e.g., Burkholderiaceae (Betaproteobacteria), Caulobacteraceae and Sphingomonadaceae (Alphaproteobacteria), increased in anode (Kirmizakis et al., 2019). They transfer electrons and promote anaerobic oxidation of PAH. The Actinobacteria genus *Nocardioides* could be involved in the power output in a soil MFC (Yu et al., 2019). The relative abundance of *Anaerolinea* (Chloroflexi) and *Nautella* (Alphaproteobacteria) increased in the anode biofilm of a plant-MFC (Liu et al., 2019). These taxonomic groups actively take part in the electron transfer in the aerobic/anoxic micro-environment around the anode. There must be unidentified electrogenic and PAH degrading strains in both anode and planktonic parts of MFC.

### Reduced Microbes in Anode and Archaea Community

In PAH degrading BES, taxonomic groups without competitive advantage will decline in abundance or even be extinct. The MFC microbial community’s resilience can be understood by studying the actual resource competition network. Spirochaetaceae (6.7%) and methanogens (e.g., *Methanosaeta* (3.1%) and *Methanobacterium* (12.5%)) in the initial inoculum disappeared in the later MFC running in both free and biofilm parts (Sharma et al., 2020). The abundance of methane producing archaea (*Methanolinea*, *Methanobacterium*, *Methanoregula*, and *Methanosaeta*) and *Candidatus* dramatically reduced after SMFC treatment for PAH removal, and the methane production could be impeded by electricity generation (Xu et al., 2019). The fermentation products, e.g., acetate and formate, are essential resources for both electroactive microbe and methanogen (Arends et al., 2014), thus there is struggle for existence. In the MFC degradation of phenol, EET relevant cytochrome c OmcA of anode microbes was significantly activated by the exogenous acetate (Shen et al., 2020), thus acetate could also be salient co-substrate in PAH elimination. The acetate is also generated by PAH degradation and utilized by exoelectrogens, thus driving the PAH dissipation.

In different BESs, the electrical current can be produced by microorganisms of all three domains of life, and electrons can be transferred to the anodes (Logan et al., 2019). However, the performance of archaea in PAH targeting MFC is seldom reported. A high throughput amplicon sequencing of archaea in a soil MFC resulted in 50,794–92,928 valid tags with 277–288 bp average length (Li et al., 2019a). Compared with the control, the species number and archaea community richness were reduced by WB biochar, whereas they were significantly increased by CB biochar. The unique OTUs were significantly increased by SB (174) and CB (514) as compared with control (120). There were 859, 820, and 750 shared OTUs between control and CB soil, control and SB soil, and control and WB soil, respectively. The phylum Euryarchaeota predominated (85–92%), followed by Thaumarchaeota (1–2.4%). The CB, SB and WB amendment dramatically reduced the Thaumarchaeota by 32, 54, and 61% respectively. The class Methanomicrobia (Euryarchaeota) was decreased by CB and WB, and Halobacteria was increased by SB. *Methanosarcina* (6–12%) and *Methanoculleus* (5–7%) were most abundant genera, followed by *Halogranum* (2–4%) and *Halovivax* (2–3%), etc. When CB and WB were added respectively, the abundance of *Methanosarcina* was decreased by 40-45%. The biochar addition also significantly altered the abundance of many other genera. The abundance of *Methanolobus chelungpuianus* in the control soil was much higher than that of biochar treated soil, therefore it could be used as a biomarker of control soil. *Methanoculleus* (phylum: Euryarchaeota; class: Methanomicrobia) and *Halobacteria* (Euryarchaeota; class: Halobacteria) could participate in the PAH degradation after biochar treatment (Li et al., 2019a). *Methanoculleus* plays an important role in the methanogenic metabolism of PAHs (Berdugo-Clavijo et al., 2012). Some methanogenic archaea genera predominated the ANT- and PHE-degrading culture (Ye et al., 2019).

### Forming a Functional Unit by Extraneous Factors

**FIGURE 4:**
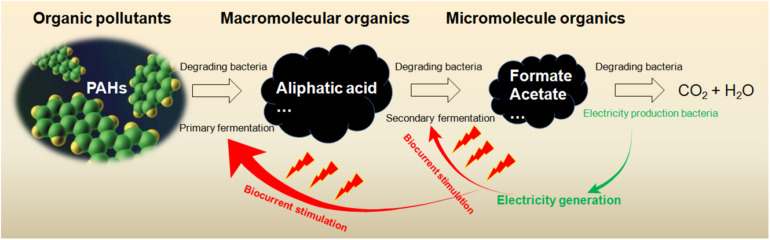
PAH degradation processes involving diversified microbes.

The surfactants induced the increase of Proteobacteria, Actinobacteria, Acidobacteria, Firmicutes, Bacteroidetes, Chloroflexi, and Planctomycetes (93–99% of total) in soil MFC (Li et al., 2018). The microbial selectivity of anionic surfactant sodium dodecyl sulfate (SDS) was the strongest; Alphaproteobacteria and Gammaproteobacteria were reduced by SDS and β-cyclodextrin, and Clostridia was increased. *Bacillus* was increased by SDS, CTAB, and β-cyclodextrin in the MFC. A metabolic network of microbes in soil MFC is revealed by the close correlations among *Bacillus*, *Solibacillus* (Firmicutes) and *Phenylobacterium* (Alphaproteobacteria) (correlation coefficient 0.9162-0.9577), and among *Alcaligenes* (Betaproteobacteria), *Sedimentibacter* (Firmicutes) and *Dysgonomonas* (Bacteroidetes) (0.9538–0.9966). Such correlations might ensure the capacity of community to make full use of limited resources (Figure 4). The generalists and specialists within the community might struggle for common resources, and the odds of survival and function of community member could be partially determined by the niche overlap. Fitness is a relational attribute (Papale et al., 2020) and could be modified by extraneous chemical reagents.

## Electrochemistry

### Electrochemical Activity

In present studies, the conventional bioelectrochemical techniques are still commonly adopted for comparison and standardization between laboratories (Yee et al., 2020). For example, the electrochemical activity of microorganisms can be well quantified by cyclic voltammetry (Zhou et al., 2020). Results showed that as strong antioxidant, the electrochemical activity of HMW PAHs was inferior to NAP, a bicyclic PAH with better bioavailability. In addition, the performance of bioelectrochemical systems is determined by the resistance properties, and thus the representation of different component resistance is also commonly applied. In a SMFC treatment of PAH pollutants, a much higher voltage was attained than that of the treatment without PAH involvement (202 vs. 176.8 mV; Xu et al., 2019), suggesting that PAHs take part in the power output when the sulfate and anode were the electron acceptors in phases I and II respectively. The circuit components solution resistance (*R*_s_) and charge-transfer resistance (*R*_ct_) should be taken into account as they could significantly impact on the MFC performance. When more β-cyclodextrin was supplemented, the *R*_s_ and *R*_ct_ values of plant-MFC anodes substantially decreased (Zhao et al., 2019), ending with the lowest resistance (1.4 and 16.5 Ω, respectively) at 5 mmol/kg. The mobility of oil and other hydrophobic substances in solution is improved by β-cyclodextrin, so is the electrochemical activity of anode biofilm, thus resulting in lower R_s_ and R_ct_ respectively (Li et al., 2015). As above-mentioned, the sand or carbon fiber amendment also decreased the MFC resistance and increased the electrochemical activity of system.

The electrode interval is also a key factor in determining the electrochemical activity of MFC. The depth of anode embedded in the soil is often dependent on the designed electrode interval (Yu et al., 2017). The oxygen decreases with the increased soil depth, and the distribution of electron acceptors, e.g., CO_2_, sulfate, nitrate, nitrite, and metal oxides, also varies in different depths of anode. Substantially, different groups of anaerobes actively participate in resource allocation, electron transfer and PAH transformation in terms of the relatively stable horizontal gradient within soil (An et al., 2013). The anode performance of different depths is closely related with the metabolic pathways of different soil anaerobes. It is problematic that the deeper the soil, the lower the redox potential, which impedes the electron transfer and PAH oxidative transformation on the anode. Moreover, the mass transfer and ohmic losses are significantly impacted by the electrode interval, and too much spacing may impair the voltage output and thus electrochemical activity of MFC (Moon et al., 2015). When the electrode spacing was 4, 6, 8, and 10 cm, the maximal voltage 294, 249, 241, and 230 mV were obtained respectively in closed SMFC reactors (Yu et al., 2017); the maximal power density (PD) was the highest and internal resistance (IR) was the lowest (12.1 mW/m^2^ and 470 Ω respectively, Supplementary Table 1) with the minimum spacing, suggesting that small electrode interval reduces the IR and favors the electron transfer. The smaller the electrode interval, the thinner the soil layer, and the less the resistance to both the mass transfer of catabolic substrates and products and the electron/proton transfer. In a traditional membrane-less MFC, the anode and cathode can be installed very closely, as long as there is no short circuit (Jang et al., 2004), so as to generate more current. In a single chamber MFC, decreasing the electrode interval from 4 to 2 cm led to the increase of power by 60% (Liu et al., 2005). In a series of soil MFCs, when cathodes are fixed, the deeper the anode is embedded, the greater the IR (An et al., 2013). In a SMFC, increasing the electrode interval from 12 to 100 cm decreased current density (CD) from 11.5 to 2.11 mA/m^2^ and PD from 1.01 to 0.37 mW/m^2^ (Hong et al., 2009).

### Power Density and Current Density

**FIGURE 5:**
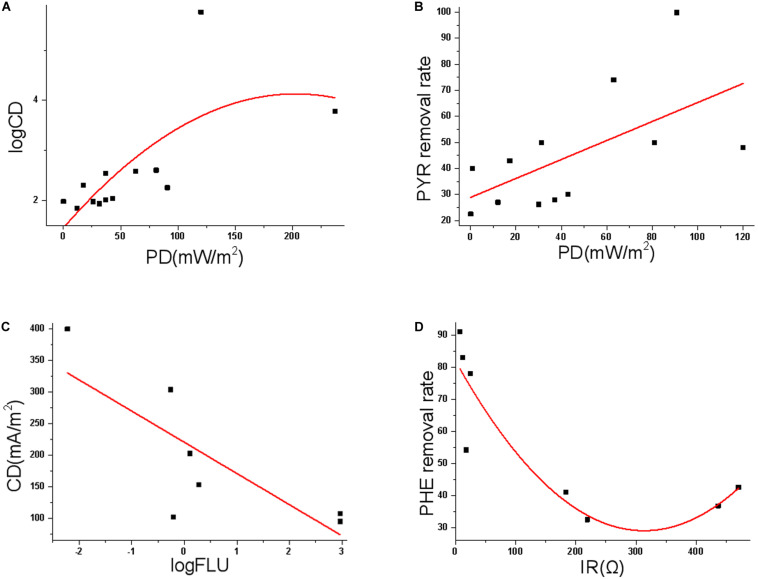
**(A)** Polynomial fit of the correlation between PD and logCD; **(B)** linear fit of the correlation between PD and PYR removal rate; **(C)** linear fit of the correlation between log(FLU start concentration) and CD; **(D)** polynomial fit of the correlation between IR and PHE removal rate.

The amount of power (product of voltage and current) per unit volume/surface area is called PD, while the magnitude of CD is the electric current per cross-sectional area at a given point in space. The MFC removal of PAH is investigated in solution, sediment, and soil environments (Kronenberg et al., 2017), and PD and CD are most commonly used indices to quantify the MFC performance (e.g., Li et al., 2019b; Liang et al., 2020; Sharma et al., 2020) (Figure 1C). For example, in a MFC study removing 100 mg/L benzene and PHE (Adelaja et al., 2014), PD ranged between 0.72 and 1.25 mW/m^2^, and the pollutant removal was more than 90% after 60-day treatment. In MFC studies treating TPH and diesel, the PD was 20 (maximal CD ∼198 mA/m^2^) (Mohan and Chandrasekhar, 2011) and 32 mW/m^2^ (Morris et al., 2009), respectively. In a MFC remediation of TPH-polluted saline-alkali soil (Li et al., 2016b), CD was 304 mA/m^2^ (Supplementary Table 1). In a 60 mL dual-chamber MFC, PHE was brush coated on the carbon cloth anode, and the maximal PD 19.2 mW/m^2^ (maximal CD 250 mA/m^2^, 20 mg PHE/cm^2^) and 37 mW/m^2^ (350 mA/m^2^, 2 mg/cm^2^) were achieved (Sharma et al., 2020). These results are somewhat not comparable, as the low concentration of PAHs (0.25 mg/L) in the MFC may lead to low PD (Zhou et al., 2020). Yet, possibly due to the high heterogeneity of MFCs designed and utilized by different groups, as well as distinct experiment conditions, no statistically significant correlation between PAH start concentration and PD/CD is identified based on recent MFC results. The start concentration of each PAH is also not significantly correlated with IR and PAH removal rate. Intriguingly, our correlation analyses suggest some substantial links (Figures 5A–D). For example, PD is positively correlated with logarithm of CD (Adj. *R*^2^ 0.419, *p* 0.026), regardless of whatever PAH; PD is also linearly and positively correlated with PYR removal rate (Adj. *R*^2^ 0.305, *p* 0.036); the logarithm of FLU start concentration is negatively correlated with CD (Adj. *R*^2^ 0.546, *p* 0.035), while IR is significantly correlated with PHE removal rate (Adj. *R*^2^ 0.751, *p* 0.013). The optimal degradation condition might be PAH specific, while operation parameters such as PD, CD and start concentration should be carefully optimized to maximize degradation of each PAH.

The performance of different MFCs can be evaluated by comparing their maximal PD. In MFCs remediating PAH-polluted soil, the maximal PD under aerobic conditions was 8.67 mW/m^2^ (Sherafatmand and Ng, 2015). In SMFCs with different depths of embedded anode (An et al., 2013), the maximal PD was 14.5 mW/m^2^. After MFC improvement with carbon fiber, the maximal PD increased to 17.3 mW/m^2^ (Li et al., 2016c), and the anode modification with bentonite–Fe further increased the maximal PD to 29.98 mW/m^2^ in a soil MFC (Yu et al., 2019). In the soil MFC, the higher PD can be obtained by modifying the anode with activated carbon (Huang et al., 2011; Li et al., 2016a) or platinum catalyst (Cao et al., 2015), but the cost of these two materials was also higher. In MFC reactors, the pre-enriched and pre-developed biofilm anodes, as compared to freshly inoculated anodes, enhanced the degradation of diesel and can be used to cope with the diesel of 8000 mg/L (Venkidusamy et al., 2016). The maximal CD and PD were 114.5 mA/m^2^ and 38.02 mW/m^2^ respectively with former two anodes, which are much higher than those of freshly inoculated anodes (49.0 mA/m^2^, 6.85 mW/m^2^). In our opinion, the PD representation based on the volume density and pollutant concentration is more applicable, since this expressive method more focuses on the actual effect of pollutant removal.

Many MFC studies use 1 kΩ external resistance (Li et al., 2016a; Xu et al., 2019) for higher CD; however, 10 kΩ might be better in simulating the operation of a larger field scale system (Kirmizakis et al., 2019). Since the peak value of polarization curve was around 10–100 kΩ, a very low IR is not necessary; the degradation capability of MFC, instead of the electricity generation, is most desired. In the scale-up of BES, the increase of electrode size and surface area, plus the resistance of wiring and connection, may cause higher ohmic resistance and the subsequent large potential loss. Selecting very low resistance in field applications may lead to impractical assessment of electricity generation. A low resistance load may result in a higher CD, which could reduce the observed voltage of MFC. With the focus of PAH removal, the high external resistance can be utilized to monitor the performance of MFC instead of maximizing the power.

### The Effect of Anode Modification

Modifying the anode surface with carbon nanomaterials, e.g., GR, GO, and CNT, at a low intensity significantly increased the surface area with high electrochemical reactivity in SMFC (Liang et al., 2020). There might be better EAB mediated EET on GR and CNT anodes within a certain potential range, as higher voltage and CD responses (9.34 and 6.75 mA/cm^2^ in GR and CNT respectively) and scan areas were observed. Additionally, in a groundwater MFC, the GAC anode, as compared with the glass bead anode, led to higher power and current output with the increase of average output to 10–20 μV (Kirmizakis et al., 2019). The higher surface area of GAC is conducive to decreasing the resistivity and increasing the electrical response of BES, and other electrodes of high conductivity and high surface area have similar strengthening effects (Tursun et al., 2016).

The PAH degradation takes place on the anode via oxidative reaction, and the anode modification cannot be overemphasized. The presence of oxidation peak, rather than reduction peak, could increase the activity of anodic half reaction. Such an oxidation peak could be elicited by the Fe-modified materials, such as two anode modifiers Fe_3_O_4_ and bentonite-Fe (Yu et al., 2019). As compared with GF anode, the *R*_ct_ of electrode was reduced by them, the anode electron transfer was promoted, and the structure of anode surface was optimized, so as to significantly increase the electricity generation (Lu et al., 2013). In light of the excellent property of ZVI, bentonite–Fe might be superior to Fe_3_O_4_. An anaerobic environment can be created on the anode via the strong reducibility of ZVI, so that more unconsumed electrons can be accepted by anode to promote the initiation of MFC and obtain a strong electrochemical activity.

In fact, the electrochemical activity of cathode is as noteworthy as that of anode, since the overall performance of bioelectrochemical system depends on both electrodes. If the cathode activity is insufficient, the anode robustness is difficult to achieve.

## Conclusion and Prospects

**FIGURE 6:**
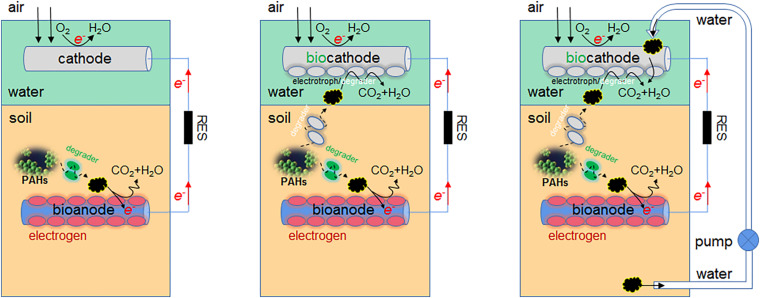
Application prospect of biocathode in PAH degradation.

In this article, firstly the PAH, especially HMW PAH, degradation in MFCs of different setups is summarized and discussed, which benefit the future decision-making in design improvement; then the taxonomic diversity and functional diversity of microbial communities in MFCs are reviewed, including both PAH degrading microbes and exoelectrogens, which is followed by the discourse of electrochemical performance of various MFCs. The anode modification/improvement is highlighted in both PAH degradation and electrochemical optimization, while the biocathode is less studied (Figure 6). In MFC, the aerobic cathode area is useful in further mineralizing degradation intermediates of azo dye to innocuous products (Oon et al., 2020), thus the auxiliary effect of cathode biofilm on PAH detoxification should also be scrutinized. Currently, the high-throughput amplicon sequencing of 16S rRNA is commonly used to analyze the abundance and diversity of microbial communities in MFC, which has replaced the traditional PCR-DGGE and T-RFLP. However, the PCR bias cannot be avoided and the information about microbial function is not provided. The shotgun metagenomic sequencing (Hao et al., 2018b), complementary to the amplicon sequencing, could more objectively reflect the microbiome *status quo* and dynamics of anode biofilm, and should be utilized in the future study.

The tentative use of MFC in PAH degradation has just arisen in recent decade. Most studies are limited in laboratory scale, and there are still numerous knowledge gaps in PAH degradation of MFC. For example, the respective role of electrochemical degradation and microbial degradation has not been distinguished clearly. PAHs in contaminated sites can be removed by adsorption and/or degradation, albeit distinct mechanisms. How to exert the synergistic effect of these three is still elusive. In the future research design, more corresponding control groups need to be set up. As for microbial degradation of PAH, there is a lack of studies about fungi and their interactions with bacteria and archaea in BES (Li et al., 2019b). The algal biofilm formed on the cathode of MFC enhanced the removal of stubborn organic matter in landfill leachate (Elmaadawy et al., 2020). What are the possible roles of protist (algae, protozoa) and metazoan (earthworm, nematode) in microbial degradation of PAHs? Recent BES studies have revealed numerous and highly diverse microorganisms capable of swapping electrons with electrodes. Electron exchange between microorganisms is also very common. So far, the interspecies electron transfer (IET) is investigated with a focus on syntrophy (Kronenberg et al., 2017), but the competitive or parasitic behavior could also be promoted by electron exchange between microbes (Moscoviz et al., 2020). Such non-mutualistic crosstalks could be universal and essential for microbial survival, and complex microbial communities in BES could be deeply influenced by them. There is still a long way to go in the MFC study of symbiosis, interaction, action mechanism, and potential application in PAH degradation. In view of the wide diversity of electroactive taxa, what are the evolutionary mechanisms of their EET capability? Clues can be collected and collated from the detailed genomic and transcriptomic characterization. Biomolecular markers of electroactivity could be identified via transcriptomics and proteomics, so as to predict or even adjust the potential of heterotroph species to accept/donate extracellular electrons. PAH elimination, organic matter conversion and power generation could be boosted simultaneously in contaminated sites if it is known to what degree the non-mutualistic IET molds the microbial community composition and structure in natural habitat and MFC.

The field of community ecology is composed of numerous theories, concepts and models, but which is applicable in MFC study? Is it feasible to establish a unified theory of ecological communities in BES? What related discipline could be a navigating framework? Understanding of cross temporal and spatial biodiversity and variation combination modes is highlighted by both community ecology, evolutionary biology and population genetics (Vellend, 2016). For novel microbial insights into PAH removal in BES, some evolution and population concepts could be borrowed to integrate contrasting views of community ecology. A theory of ecological communities tailored for BES environment could be based on a few paramount processes, e.g., selection among species, gene flow/dispersal, genetic drift, and mutation, etc. More specific models describing the dynamics of microbial communities in MFC could be validated experimentally. Based on these models the impacts of many processes, e.g., colonization, stress, competition, facilitation, succession, and local extinction, could be better understood and applied extensively for maximizing the efficacy of ecological communities in BES removal of PAHs. The new path for musing on biological composition and diversity in MFC should be provided and the novel approach for enhancing pollutant degradation *in situ* and/or *ex situ* could be developed.

## Author Contributions

D-CH, P-GX, and L-FW conceived the topic and content. D-CH and X-JL searched the literature, analyzed the data, and wrote the manuscript. All authors provided the fund support, interpreted the results, critically revised the manuscript for important viewpoints, and approved the final manuscript.

## Conflict of Interest

The authors declare that the research was conducted in the absence of any commercial or financial relationships that could be construed as a potential conflict of interest.
