# Supplementary material for: The Utility of Electrochemical Systems in Microbial Degradation of Polycyclic Aromatic Hydrocarbons: Discourse, Diversity and Design
Source: Front Microbiol. 2020 Oct 23;11:557400. doi: 10.3389/fmicb.2020.557400 (PMC7644954; doi:10.3389/fmicb.2020.557400)
Supplement: Supplementary file 1 [file Data_Sheet_1.docx]

Table S1 Examples of MFCs used in degrading PAHs

| Reference | Type of BES | Environment/Matrix | Pollutant | Start concentration | Removal rate(%) | Control  (PAH removal,%) | Experiment duration  (days) | Max.current density  (mA/m2) | Max.power density  (mW/m2) | Anode | Cathode |
| --- | --- | --- | --- | --- | --- | --- | --- | --- | --- | --- | --- |
| Liang et al.2020 | SMFC | sediment | PHE,PYR | 1.23(PHE),1.15(PYR)mg/kg | PYR:graphene oxide(GO)-SMFC(69.6),  graphene(GR)-SMFC(68.2),carbon nanotube(CNT)-SMFC(66.7);  PHE:CNT(78.1),GR(73),GO(71.2) | graphite felt(GF)-SMFC:42.3  (PYR),45.6(PHE) | 110 | GR:93400(forward scan),-121800(reverse scan);CNT:67500  (forward scan),-85000  (reverse scan) | GR-SMFC,0.98±0.14kJ;  GO-SMFC,  0.87±0.04kJ;CNT-SMFC,  0.57±0.06kJ;GF-SMFC,  0.49±0.07kJ | GR,GO,CNT,GF | GF coated  with Pt/C catalyst |
| Sharma et al.2020 | 60 mL dual chamber MFC,PEM:NafionTM 117 membrane | slurry brush coated on carbon cloth | PHE | 2 and 20mg/cm2 | nearly 100,validated by FTIR | anode without PAH load | 50 | 350(2mg/cm2);  250(20mg/cm2) | 37(2mg/cm2);  19.2(20mg/cm2) | carbon cloth anode  loded with PAH | Pt/C-coated carbon cloth |
| Zhou et al. 2020 | 100 mL single-chamber MFC | synthetic media | PHE,PYR,NAP | 0.25mg/L | 89.2(PHE),51.4(PYR) in NAP-PHE(1:2) and  NAP-PYR(1:4),respectively | no NAP:42.9(PHE),  22.0%(PYR) | 20(domestication),  120 h(degradation) | 199 | 0.0171±0.004(PHE);  0.0641±0.0012(PYR);  0.0473±0.001(NAP-PHE);  0.1586±0.0044(NAP-PYR);  0.0246±0.007(PHE-PYR)  mV/m2 | carbon cloth | air-cathode,carbon cloth  loaded with Pt/C catalyst |
| Li et al.2019a | MFC | soil | 16 priority PAHs | 24.9mg/kg | with chicken manure(CB):68(total PAHs),74(PHE),60(ANT),79(FLU),68(PYR),67(BaA),  50(CHR),54(BbF),50(BkF),73(BaP),50(IcdP),  25(DBah),28.6(BghiP) | total PAHs:39,PHE:42,  FLU:43,PYR:26,  CHR:30,BBF:9,BKF:25,BAP:45  (wood sawdust,WB);total PAHs:39,PHE:30,FLU:59,PYR:50,  CHR:33,BbF:18,BkF:25,BaP:9  (wheat straw,SB) | 223 | NR | 233.7±4.4mV(no biochar);  682 C(WB) | one-layer carbon mesh | rolling air-cathode  (activated carbon catalyst  layer,carbon black gas  diffusion layer,steel  stainless mesh current  collector) |
| Li et al.2019b | 600-mL traditional “H” two-cell MFC | diesel | diesel hydrocarbon | 3.26 g/L diesel | 50% diesel during 8 d | no E2 strain | 520 h | 86.43 | 31.37 | carbon cloth  (6cm×7cm) | carbon cloth  (6cm×7cm) |
| Liu et al. 2019 | plant-driven SMFC | sediment | PHE,PYR | 1.4(PHE),1.29(PYR)mg/kg | 62.98(PHE),57.02(PYR) | plant without anode column:  PHE 47.7,  PYR 43.1;anode  without plant/column:  PHE 42.9,PYR 41.8 | 82 | NR | 260mV | separated sand-filled  anode(graphite felt)  column | graphite felt  cathode coated  with Pt catalyst |
| Yu et al. 2019 | MFC | soil | ANT,PHE,PYR | 219(ANT),331(PHE),  98(PYR)mg/kg | 36.62(ANT),32.48(PHE),26.24(PYR) | graphite felt(GF) anode:  29.95(ANT),  24.14(PHE),  21.62(PYR);GF+Fe3O4:  31.56(ANT),  28.53(PHE),23.28(PYR) | 45 | 0.16A | 29.98 | graphite felt+  bentonite-Fe | air cathode |
| Zhao et al. 2019 | single-chamber plant-MFC | soil | PHE,PYR | 1000mg/kg | 5mmol/kg β-cyclodextrin:54.2(PHE),48.4(PYR) | 5mmol/kg Tween 80:  45.5(PHE),41.4(PYR) | 55 | NR | 184.9mV | graphite felt | graphite felt |
| Kirmizakis et al. 2019 | MFC | groundwater | NAP,methyl NAP;aromatic C12-C16,C16-C21,C21-C35 | TPH 1546mg/L(aromatic  C12-C16:131.55,C16-C21:  22.21,C21-C35:8.6mg/L) | anode with granular activated carbon:C12-C16,99.49;C16-C21,99.86;C21-C35,97.08 | anode with glass beads:  C12-C16,46.12;  C16-C21,62.63;  C21-C35,94.65 | 21 | 20 | 750nW | graphite chamber | porous graphite  base plate |
| Wang et al. 2019c | tubular MFC | soil | TPHs | NR | 48-59(saturated sandy soil) | 42-45(clay soil) | 248 | 58 | 120 | carbon felt | carbon cloth  air cathode |
| Wang et al. 2019a | constructed wetland-MFC | Typha orientalis wetland,synthetic municipal wastewater | PHE,ANT | 0.17(PHE),0.17(ANT)mg/L  synthetic wastewater | PHE:95.3±0.9(CFF-nZVI);ANT:96.5±1.5(CFF-nZVI) | PHE:88.4±2.9(FN),  93.6±1.6(FN-nZVI),  93.6±1.6(CFF);ANT:  92.5±1.2(FN),  95.2±1.0(FN-nZVI),  95.8±1.5(CFF) | 182 | 94.3(CFF-nZVI) | 26(CFF-nZVI) | FN,FN+nZVI,CFF,  CFF+nZVI | similar to  corresponding anode |
| Xu et al.2019 | SMFC | sediment | PYR,PHE | 10mg/kg dry sediment | PYR:79.4,PHE:88.2(MFC+macrophyte) | PYR:29.1,PHE:35.4  (no MFC and plant) | 65 | NR | 202mV | three pieces of modified polyacrylonitrile-based  graphite felt | air cathode |
| Zhao et al. 2018 | cylindric SMFC | river sediment | PHE,PYR,CHR | NR | methanol stimulation:87(PHE),70(PYR),65(CHR) | no methanol stimulation:37-55  (anode district sediment),28-50  (non-electrode district sediment) | 200 | 0.2 mA | 0.4V | NR | NR |
| Li et al.2018 | connected MFC | soil | 16 priority PAHs | 2.4(PHE),1.9(FLU),1.6(PYR),  1.65(CHR),1(BbF),0.9(BkF)mg/kg | 90(lecithos,SDS,CTAB,β-cyclodextrin) | 15-60 removal(without  lecithos,SDS,CTAB,  β-cyclodextrin) | 182 | 153.8±0.1  (lecithos) | accumulated charge  output 5846 C  (lecithos) | one-layer carbon  mesh | activated carbon  air-cathode |
| Hamdan et al.2017 | marine SMFC | marine sediment | NAP,2-methyl NAP,PHE | PHE:20mg/kg dry sediment | PHE:93.83±1.68 | 40.37±3.24(open circuit) | 196 | NR | 0.57mV | carbon fiber brushes | carbon fiber brushes |
| Yu et al.2017 | closed MFC,electrodes spaced at 4 cm | soil | 20 PAHs | 103±6.4(ANT),94.1±2.4(PHE),  82.5±1.7(PYR)mg/kg | 54.2±2.7(ANT),42.6±1.9(PHE),27.0±2.1(PYR) | open MFC:20.8±1.1(ANT),  17.3±1.2(PHE),  11.7±0.9(PYR) | 175 | 70 | 12.1 | activated CFF | activated CFF |
| Yan et al. 2017 | SMFC | sediment | BaP | 1mg/kg dry sediments | 92 | 54(natural attenuation) | 970 | 0.19±0.08mA  (average) | 19.8 | graphite felt cylinder  (6.4×8×0.5cm3) | round graphite felt  (9.5×0.5cm2) |
| Li et al. 2017a | MFC | synthetic wastewater | BbF,BkF,BaP | 3.5(BbF),12.5(BkF),  14.5(BaP)mg/kg | 74(sediment close to anode) | 60(away from anode) | 72 | 380 | 63±3 | carbon mesh with honeycomb-structure  supports | rolling activated  carbon air-cathode |
| Li et al. 2017b | SMFC | river sediment | 12 PAHs | BaP,BkF>12mg/kg | 50(BaP),50(BkF) | BaP:37.4(S Control),  30.8(W Control);  BkF:28(S Control),  21.8(W Control) | 60 | 400 | 81 | 3-D anode (Tri-DSA) with honeycomb structure of carbon cloth+supporting skeleton | floating air cathode |
| Li et al.2016a | MFC | saline soil | TPHs,n-alkanes,16 priority PAHs | 1963.49(PHE),282.32(ANT),  919.13(FLU),945.06(PYR),  306.12(BaA),1112.94(CHR),  411.74(BbF),101.96(BkF),  168.36(BaP),119.43(IcdP),  116.41(DBah),  215.58(BghiP)mg/kg | PHE,78;ANT,73;FLU,48;PYR,30;BaA,42;  CHR,12;BbF,20;BkF,34;BaP,28;IcdP,30;  DBah,21;BghiP,40 | NR | 135 | 108 | 43 | carbon mesh | activated carbon  air-cathode |
| Li et al.2016b | MFC | saline-alkali soil | TPHs,n-alkanes,16 priority PAHs | 0.484mg/kg(PAHs) | PHE,83;ANT,90;FLU,94;PYR,90;BaA,86;  CHR,85;BbF,89;BkF,88;BaP,84;IcdP,83;  DBah,85;BghiP,82 | NR | 65 | 304 | NR | carbon mesh | activated carbon  air cathode |
| Li et al.2016c | MFC | soil | 16 priority PAHs, 30 n-alkanes | 7.942±7.476mg/kg (PAHs) | 37(16 PAHs),PHE:41,ANT:66,FLU:45,PYR:43,  BaA:52,CHR:36,BbF:30,BkF:31,BaP:41,  IcdP:40,DBah:35,BghiP:34 | NR | 144 | 203 | 17.3 | graphite rod | activated carbon  air-cathode |
| Li et al.2015 | MFC | aged soil | TPHs,n-alkanes,16 priority PAHs | same as Li et al. 2016a | 50(16 PAHs),ACE:91,DBah/BghiP:22.5±1.5 | NR | 135 | 0.28/g soil | 2.76×10-4/g soil | carbon mesh | activated carbon  air-cathode |
| Zhang et al.2015 | MFC | soil | TPHs,16 priority PAHs,C8-C40 n-alkanes | 25700 mg/kg dry soil,PHE,  FLU,PYR,CHR,BbF > 300 ng/g | 14(horizontal anodes,HA);24.6(PHE),  4.6(FLU),8.5(PYR),  10.4(CHR),9.4(BbF) | 8.3(vertical anodes,VA),  6.4(open circuit) | 135 | NR | HA max.0.282V,  VA max.0.285V | acetone-cleaned  carbon mesh | air-cathode:stainless  steel mesh with  catalyst layer  (activated carbon)  on soil side and  gas diffusion layer  on air side |
| Yan et al.2015 | SMFC | sediment | PYR,BaP | 2 mg/kg dry sediment (BaP),  4 mg/kg dry sediment(PYR) | PYR:87.18 ± 5.62(macrophyte+SMFC),  BaP:76.40 ± 6.93 | PYR:27.03±4.06(control),  BaP:14.29±9.64 | 367 | 17000 | 61-65mV | graphite felt | graphite felt |
| Li et al.2014 | MFC | saline soil | C8-C40 n-alkanes,  16 PAHs | 5.653 mg/kg(total PAHs) 609 mg/kg(n-alkanes) | 36(PAHs),PHE:40,ANT:34,FLU:45,  PYR:28,BaA:44,CHR:7,BbF:19,  BkF:45,BaP:30,IcdP:23 | NR | 180 | 102 | 37 | carbon mesh | activated carbon  air-cathode |
| Wang et al.2012 | MFC | saline soil | TPHs,16 priority PAHs(PHE,CHR,PYR representing 75%) | 0.5-0.8 mg/kg soil | 15.2(TPH),PHE:55,ANT:50,FLU:54,  PYR:40,BaA:50,CHR:33,BaP:62,  DBah:33,BghiP:50 | 6.9(open circuit) | 25 | NR | 0.85 | carbon mesh | carbon mesh  air-cathode |

Current (I) = U/R, where U is the voltage difference between anode and cathode, and R is the external circuit resistance used in experiments. Power (P) = UI. Both I and P are normalized by the anode surface area. Two rings PAH: NAP, naphthalene; Three rings: ACE, acenaphthylene; PHE, phenanthrene; ANT, anthracene; Four rings: PYR, pyrene; FLU, fluoranthene; CHR, chrysene; BaA, benzo(a)anthracene; Five rings: BaP, benzo(a)pyrene; BbF, benzo(b)fluoranthene; BkF, benzo(k)fluoranthene; DBah, dibenzo(a,h)anthracene; Six rings: BghiP, benzo(g,h,i)perylene; IcdP, indeno(1,2,3-cd)pyrene. TPH, total petroleum hydrocarbon. NR, not reported. CFF, carbon fiber felt. nZVI, nano zero valent iron. FN, foamed nickel.

Table S2 Examples of microbial communities involved in PAH removal of MFCs

| Reference | No.of taxa in anode  biofilm/soil/sediment | Dominant taxonomic group (relative abundance, %) in anode biofilm/soil/sediment | Taxonomy:Kingdom/Phylum/Class | Taxonomy:Order/Family/Genus | Putative function | Decreased taxa in electrode  district/soil/sediment |
| --- | --- | --- | --- | --- | --- | --- |
| Liang et al.2020 | unique OTU:219(GF),1012(GR),  256(GO),253(CNT) | 1Pseudomonas,Thauera,Diaphorobacter,  Lysobacter,2Tumebacillus | 1Proteobacteria,2Firmicutes |  | PAH degradation | Thiobacillus |
|  |  | SEEP-SRB1 and Desulfobulbus | Class:Deltaproteobacteria |  | sulfur cycling,electron transfer |  |
|  |  | Proteobacteria(29.05),Chloroflexi(13.89),  Acidobacteria(6.4),Actinobacteria(5.96),  Bacteroidetes(4.66),Planctomycetes(4.48),  Firmicutes(4.1) |  |  |  |  |
|  |  | GR/GO:Betaproteobacteria(15.7-16.9),  Deltaproteobacteria(13-13.9),  Anaerolineae(9.9-13),  Gammaproteobacteria(7.3-12.8) |  |  |  |  |
|  |  | GF/CNT:Betaproteobacteria(21.9-22.2),  Deltaproteobacteria(14.1-15.2),  Gammaproteobacteria(11.2-11.3),  Anaerolineae(8.8-10.4) |  |  |  |  |
| Sharma et al.2020 | 210 | Ralstonia | Class:Betaproteobacteria | Family:Burkholderiaceae | PAH degradation | Spirochaetaceae,methanogens  (Methanobacterium,Methanosaeta) |
|  |  | Pseudomonas | Class:Gammaproteobacteria | Family:Pseudomonadaceae | assimilate and adsorb phenanthrene, produce biosurfactants, and modify cell surface hydrophobicity for better attachment to hydrocarbons;generating phenazines, pycocyanins,and biofilms for EET |  |
|  |  | Rhizobium | Class:Alphaproteobacteria | Family:Rhizobiaceae | promoting gene expression of hydrocarbon-metabolizing enzymes such as alkane |  |
|  |  | Thauera | Class:Betaproteobacteria | Family:Zoogloeaceae | monooxygenase and naphthalene dioxygenase,production of siderophores, phytochelatins, amino acids, and other organic acids |  |
|  |  | Rhodococcus | Phylum:Actinobacteria | Family:Nocardiaceae | aromatic hydrocarbon degrader |  |
| Zhou et al. 2020 | NAP-PYR:242 OTUs | Anaerolineaceae | Phylum:Chloroflexi;Class:Anaerolineae |  | degradation of benzene and hydrocarbons | NR |
|  |  | Clostridium | Phylum:Firmicutes;Class:Clostridia |  | PAH degradation |  |
|  |  | Roseiflexus | Class:Chloroflexi;Family:Roseiflexaceae |  | NR |  |
|  |  | Cyanobacteria |  |  | PAH degradation |  |
|  |  | Methylophilus | Class:Betaproteobacteria;Order:Nitrosomonadales; | Family:Methylophilaceae | PAH degradation |  |
|  |  | Chitinophagaceae | Phylum:Bacteroidetes;Class:Chitinophagia |  | use NAP, PHE, and PYR as carbon source |  |
| Kirmizakis et al. 2019 | 26 phyla,143 families,209 genera | Proteobacteria,Bacteroidetes,Firmicutes |  |  |  | NR |
|  |  | Psuedomonadaceae | Class:Gammaproteobacteria | Order:Pseudomonadales | aerobic hydrocarbon degrader;utilize electron shuttles to transfer electrons to anode |  |
|  |  | Rhodocyclaceae | Class:Betaproteobacteria | Order:Rhodocyclales | hydrocarbon degrader |  |
|  |  | Comamonadaceae | Class:Betaproteobacteria |  | hydrocarbon degrader |  |
|  |  | Caulobacteraceae | Class:Alphaproteobacteria |  | Fe(III) reducer,electron transfer;  enhanced anaerobic oxidation of organic contaminant |  |
|  |  | Burkholderiaceae | Class:Betaproteobacteria |  | Fe(III) reducer,electron transfer;  enhanced anaerobic oxidation of organic contaminant |  |
|  |  | Sphingomonadaceae | Class:Alphaproteobacteria | Order:Sphingomonadales | Fe(III) reducer,electron transfer;  enhanced anaerobic oxidation of organic contaminant |  |
|  |  | Novosphingobium | Class:Alphaproteobacteria | Family:Sphingomonadaceae | Fe(III) reducer,electron transfer;  enhanced anaerobic oxidation of organic contaminant |  |
| Li et al.2019a | bacteria OTUs 3662(WB),4019(no biochar);archaea OTUs 1224(SB),1623(CB),980(WB),  1117(no biochar) | Actinotalea(with CB/SB) | Phylum:Actinobacteria;Order:Micrococcales | Family:Cellulomonadaceae | anaerobic PAH degradation | Thaumarchaeota,Proteobacteria,  Verrucomicrobia(with biochar) |
|  |  | Desulfatitalea(WB) | Class:Deltaproteobacteria;Order:Desulfobacterales | Family:Desulfobacteraceae | SRB;electroactive | Gemmatimonadetes,Acidobacteria,Nitrospirae,  Firmicutes,Bacteroidetes(WB) |
|  |  | Azospirillum(WB) | Class:Alphaproteobacteria;Order:Rhodospirillales; | Family:Rhodospirillaceae | electroactive | Rhodococcus(CB/SB) |
|  |  | Chloroflexi(WB) |  |  | electroactive | Nitriliruptor(SB/WB) |
|  |  | Actinobacteria,Thermomicrobia(with biochar);  Acidobacteria,Nitrospirae,Firmicutes(CB/SB) |  |  | PAH degradation | Perlucidibaca,Pseudomonas,Alcanivorax,  Altererythrobacter,  Azohydromonas,Methanolobus(biochar) |
|  |  | Mycobacterium(biochar) | Phylum:Actinobacteria | Family:Mycobacteriaceae | PAH degradation | Methanomicrobia,Methanosarcina,unidentified Halobacteriaceae,  Natronococcus(CB/WB) |
|  |  | unidentified Anaerolineaceae(biochar) | Phylum:Chloroflexi;Class:Anaerolineae | Order:Anaerolineales | PAH degradation | Natronorubrum,Halogranum(CB) |
|  |  | Rhodococcus(biochar) | Phylum:Actinobacteria | Family:Nocardiaceae | PAH degradation | Halobaculum(SB) |
|  |  | Methanoculleus(biochar) | Archaea;Phylum:Euryarchaeota;  Class:Methanomicrobia | Family:Methanomicrobiaceae | PAH degradation |  |
|  |  | Nitriliruptor(CB) | Phylum:Actinobacteria | Family:Nitriliruptoraceae | PAH degradation |  |
|  |  | Georgenia(CB) | Phylum:Actinobacteria;Order:Micrococcales | Family:Bogoriellaceae | PAH degradation |  |
|  |  | Halobacteria(SB) | Archaea;Phylum:Euryarchaeota;Class:Halobacteria | Family:Halobacteriaceae | PAH degradation |  |
| Liu et al. 2019 | 1897 common OTUs;unique OTUs:  513(with plant/anode column),  241(with plant/no column),155(with column/no plant),159(no plant/column),66(initial sediment) | Proteobacteria,Chloroflexi,  Bacteroidetes |  |  |  | NR |
|  |  | Nautella | Class:Alphaproteobacteria | Order:Rhodobacterales;Family:Rhodobacteraceae | electrochemically active |  |
|  |  | Desulfobulbus | Class:Deltaproteobacteria | family:Desulfobulbaceae | electrochemically active;organic  contaminant biodegradation |  |
|  |  | Anaerolinea | Phylum:Chloroflexi;Class:Anaerolineae | Family:Anaerolineaceae | electron transfer |  |
|  |  | Sphingobacteriia,Bacteroidetes_vadinHA17 | Phylum:Bacteroidetes |  | NR |  |
|  |  | SEEP-SRB1 |  |  | NR |  |
| Wang et al. 2019c | NR | Geobacter(bioanode) | Class:Deltaproteobacteria | Family:Geobacteraceae | electrochemically active;anaerobic  degradation of aromatic hydrocarbon | NR |
|  |  | Parvibaculum | Class:Alphaproteobacteria | Family:Rhodobiaceae | utilize oxygen diffused from air  cathode,protect anaerobic bacteria |  |
|  |  | Pseudomonas | Class:Gammaproteobacteria | Family:Pseudomonadaceae | extracellular electron transfer;  hydrocarbon degradation |  |
|  |  | Actinobacteria |  |  | hydrocarbon conversion |  |
|  |  | Bacteroidetes(in sandy soil) |  |  |  |  |
|  |  | Chloroflexi(clay soil) |  |  |  |  |
|  |  | Acetanaerobacterium,Caloribacterium(bioanode) | Phylum:Firmicutes |  | anaerobic electron transfer to the anode |  |
|  |  | Deltaproteobacteria(clay soil,biofilm) |  |  | utilize degradation intermediates;  transfer electrons to electrode |  |
|  |  | Anaeromyxobacter(bioanode) | Class:Deltaproteobacteria | Family:Myxococcaceae | utilize degradation intermediates;  transfer electrons to electrode |  |
|  |  | Telmatospirillum(bioanode) | Class:Alphaproteobacteria | Order:Rhodospirillales;  Family:Rhodospirillaceae | utilize degradation intermediates;  transfer electrons to electrode |  |
|  |  | Rhodococcus | Phylum:Actinobacteria | Family:Nocardiaceae | utilize oxygen diffused from air  cathode,protect anaerobic bacteria |  |
|  |  | Azospirillum | Class:Alphaproteobacteria | Order:Rhodospirillales;  Family:Rhodospirillaceae | utilize oxygen diffused from air  cathode,protect anaerobic bacteria |  |
|  |  | Achromobacter | Class:Betaproteobacteria | Order:Burkholderiales;  Family:Alcaligenaceae | aerobic hydrocarbon degradation |  |
|  |  | Bordetella | Class:Betaproteobacteria | Order:Burkholderiales;  Family:Alcaligenaceae | aerobic hydrocarbon degradation |  |
|  |  | Burkholderia | Class:Betaproteobacteria | Order:Burkholderiales;  Family:Burkholderiaceae | aerobic hydrocarbon degradation |  |
|  |  | Corynebacterium | Class:Actinobacteria | Order:Corynebacteriales;  Family:Corynebacteriaceae | aerobic hydrocarbon degradation |  |
|  |  | Mycobacterium | Class:Actinobacteria | Order:Corynebacteriales;  Family:Mycobacteriaceae | aerobic hydrocarbon degradation |  |
|  |  | Nocardioides | Class:Actinobacteria | Order:Propionibacteriales;  Family:Nocardioidaceae | aerobic hydrocarbon degradation |  |
|  |  | Pseudoxanthomonas | Class:Gammaproteobacteria | Order:Xanthomonadales;  Family:Xanthomonadaceae | aerobic hydrocarbon degradation |  |
| Wang et al. 2019a | OTUs 1675 - 2858 | Bacillus | phylum:Firmicutes;Class:Bacilli | Family:Bacillaceae | PAH degradation;oxidation  and reduction of nZVI | Proteobacteria,Spirochaetes(with nZVI) |
|  |  | Paludibacter | Class:Bacteroidetes | Family: Paludibacteraceae | PAH degradation;remove refractory  organic compounds anaerobically | Class:Betaproteobacteria,  Flavobacteriia(nZVI) |
|  |  | Desulfovibrio | [Class:Deltaproteobacteria](https://cn.bing.com/search?q=Deltaproteobacteria+wikipedia&FORM=LFACTRE) | Family:Desulfovibrionaceae | PAH degradation;oxidation and  reduction of nZVI |  |
|  |  | Lactococcus | [Class:Bacilli](https://cn.bing.com/search?q=Bacilli+wikipedia&FORM=LFACTRE) | Family:Streptococcaceae | PAH degradation;remove  refractory organic compounds  anaerobically |  |
|  |  | Pseudomonas | [Class:Gammaproteobacteria](https://cn.bing.com/search?q=Gammaproteobacteria+wikipedia&FORM=LFACTRE) | Family:Pseudomonadaceae | PAH degradation |  |
|  |  | Phylum:1Firmicutes,2Bacteroidetes,  3Actinobacteria(with nZVI) | Class:1Bacilli,2Bacteroidia,3Actinobacteria,  4Deltaproteobacteria(with nZVI) |  |  |  |
| Xu et al.2019 |  | 1Firmicutes,2Proteobacteria,3Nitrospirae,  4Chloroflexi,  5Bacteroidetes,6Actinobacteria | 2Betaproteobacteria,Gammaproteobacteria;4Anaerolineae; | 3Nitrospira;5Bacteroides;  6Kocuria,Mycobacterium | exoelectrogen,PYR degradation | Methanobacterium,Candidatus,  Methanoregula,Methanolinea,  Methanosaeta |
|  |  | Bacilli,Clostridia | Phylum:Firmicutes | Geobacillus | PAH degradation |  |
|  |  | Geobacteraceae sp.,Desulfobulbus,Desulfovibrio | Class:Deltaproteobacteria |  | exoelectrogen,sulfate-reducing |  |
|  |  | Methanomicrobia | Archaea;Phylum:Euryarchaeota |  |  |  |
|  |  | Gallionella,Dechloromonas | Betaproteobacteria |  |  |  |
|  |  | Pseudomonas,Nevskia | Gammaproteobacteria |  | exoelectrogen;PAH degradation |  |
|  |  | Hyphomicrobium,Rhodoplanes | Alphaproteobacteria |  | PHE degradation |  |
| Yu et al. 2019 |  | Thermomonas | Class:Gammaproteobacteria | Order:Xanthomonadales;  Family:Xanthomonadaceae | power generation | C1-B045,Iamia |
|  |  | Proteiniphilum | Phylum:Bacteroidetes;Class:Bacteroidia | Order:Bacteroidales;  Family:Dysgonamonadaceae | power generation |  |
|  |  | Nocardioides | Phylum:Actinobacteria | Order:Propionibacteriales;  Family:Nocardioidaceae | power generation |  |
|  |  | Pseudoxanthomonas | Class:Gammaproteobacteria | Order:Xanthomonadales;  Family:Xanthomonadaceae | power generation |  |
|  |  | Geobacter | Class:Deltaproteobacteria | Family:Geobacteraceae | power generation |  |
| Li et al.2018 | 21 bacterial phyla | Proteobacteria,Firmicutes,Bacteroidetes(with SDS/β-cyclodextrin),Actinobacteria/Acidobacteria(lecithos),  Chloroflexi/Planctomycetes(lecithos/β-cyclodextrin) |  |  | establish an electro-metabolic network of microbes | (SDS/β-cyclodextrin) |
|  |  | Clostridia(with SDS/β-cyclodextrin) | Phylum:Firmicutes |  |  | (SDS/CTAB/glyceryl monostearate) |
|  |  | Bacteroidia | Phylum:Bacteroidetes |  |  | Planctomyces(SDS/CTAB) |
|  |  | Clostridium(with lecithos) | Phylum:Firmicutes;class:Clostridia |  | electricity generation;PAH degradation | Aquimonas,Xanthomonadales(CTAB) |
|  |  | Bacillus(with SDS/β-cyclodextrin/CTAB) | Phylum:Firmicutes;Class:Bacilli | Family:Bacillaceae | exoelectrogens and degraders | Lachnospiraceae,Propionispora(glyceryl monostearate) |
|  |  | Clostridiales,Pseudomonas,  Syntrophomonas(with SDS/CTAB) |  |  | exoelectrogens and degraders | Perlucidibaca,Pseudomonas,  Sphingobium(lecithos) |
|  |  | Proteiniphilum(with SDS) | Phylum:Bacteroidetes;Class:Bacteroidetes;  Order:Bacteroidales |  |  | Syntrophomonas(β-cyclodextrin) |
|  |  | Lachnospiraceae(with CTAB) | Phylum:Firmicutes;Class:Clostridia;  Order:Clostridiales |  |  |  |
|  |  | Perlucidibaca,Pseudomonas,  Methylobacillus(β-cyclodextrin) |  |  |  |  |
| Zhao et al. 2018 | NR | Geobacter metallireducens | Class:Deltaproteobacteria | Family:Geobacteraceae | electroactive degrader | unclassified Rhodocyclaceae,Smithella |
|  |  | Desulfobulbus | Class:Deltaproteobacteria | Family:Desulfobulbaceae | electroactive degrader |  |
|  |  | Acinetobacter | Class:Gammaproteobacteria | Family:Moraxellaceae | PAH degradation |  |
|  |  | Nitrospira | Phylum:Nitrospirae;Class:Nitrospira | Family:Nitrospiraceae | TOC degradation |  |
|  |  | Uncultured Coriobacteriaceae | Phylum:Actinobacteria;Class:Coriobacteriia | Order:Coriobacteriales | PAH degradation |  |
| Hamdan et al.2017 | 2249 OTUs | Geoalkalibacter | Class:Deltaproteobacteria |  | exoelectrogenic;utilize iron(III) as terminal electron acceptor(TEA) | Actinobacteria |
|  |  | Desulfuromonas | Class:Deltaproteobacteria |  | sulfur reducing;oxidize acetate and other multi-carbon organic substrates | Streptomyces |
|  |  | Deltaproteobacteria |  |  |  |  |
|  |  | Alphaproteobacteria |  |  |  |  |
|  |  | Sphingomonas,Hoflea,Mesorhizobium | Class:Alphaproteobacteria |  | anode reduction |  |
|  |  | Cycloclasticus | Class:Gammaproteobacteria |  | PAH degradation |  |
|  |  | Pseudonocardia | Phylum:Actinobacteria |  | PAH degradation |  |
|  |  | Clostridium | Phylum:Firmicutes;Class:Clostridia |  | current production |  |
| Li et al.2017b |  | Deltaproteobacteria 12.26 |  |  |  |  |
|  |  | Geobacter 4.94 |  |  |  |  |
|  |  | Gammaproteobacteria |  |  | electron transfer |  |
|  |  | Flavobacteria and Bacteroidia |  |  | electricity generation |  |
| Li et al. 2017a | NR | Chloroflexi, Firmicutes |  |  | degradation of hydrocarbons | Proteobacteria,Bacteroidetes |
|  |  | Geobacter psychrophilus | Class:Deltaproteobacteria | Family:Geobacteraceae | exoelectrogenic | TM7,Actinobacteria,  Gemmatimonadetes,SR1 |
|  |  | Longilinea | Phylum:Chloroflexi;Class:Anaerolineae | Order:Anaerolineales | ferment carbohydrates | Dongia,Arenimonas,  Aquabacterium,  Ohtaekwangia |
|  |  | Clostridium | Phylum:Firmicutes;Class:Clostridia |  | current production,carbohydrates degradation | Gp7,Aquicella,Thermomonas |
| Yan et al. 2017 | NR | Firmicutes,Proteobacteria,Actinobacteria,  Bacteroidetes,Nitrospirae |  |  |  | Methanosaeta,Candidatus methanoregula,Methanosarcina |
|  |  | Bacillus | Phylum:Firmicutes;Class:Bacilli | Family:Bacillaceae | anodic electron transfer;carbon-cycling,  anaerobic organic remediation |  |
|  |  | Pseudomonas | Class:Gammaproteobacteria | Family:Pseudomonadaceae | electron transfer;carbon-cycling,organic remediation;aromatics degradation |  |
|  |  | Clostridium | Phylum:Firmicutes;Class:Clostridia |  | anodic electron transfer;carbon-cycling,  anaerobic organic remediation |  |
|  |  | Desulfobacca | Class:Deltaproteobacteria;  Order:Syntrophobacterales | Family:Syntrophaceae | anaerobic carbon degradation |  |
|  |  | Burkholderia | Class:Betaproteobacteria | Family:Burkholderiaceae | electron transfer;carbon-cycling,organic remediation |  |
|  |  | Geobacter metallireducens GS-15 | Class:Deltaproteobacteria | Family:Geobacteraceae | electron transfer;anaerobic degradation of aromatics |  |
|  |  | Methanolinea | Archaea;Phylum:Euryarchaeota;  Classe:Methanomicrobia | Order:Methanomicrobiales | methanogen |  |
|  |  | Dechloromonas aromatica RCB | Class:Betaproteobacteria | Family:Azonexaceae | anaerobic degradation of aromatic  hydrocarbon and benzene |  |
|  |  | Shewanella | Class:Gammaproteobacteria | Family:Shewanellaceae | electron transfer |  |
|  |  | Anaeromyxobacter | Class:Deltaproteobacteria | Family:Myxococcaceae | electron transfer |  |
|  |  | Rhodobacter | Class:Alphaproteobacteria | Family:Rhodobacteraceae | electron transfer |  |
|  |  | Escherichia sp. | Class:Gammaproteobacteria | Family:Enterobacteriaceae | anaerobic degradation of aromatics |  |
|  |  | Sphingomonas wittichii RW1 | Class:Alphaproteobacteria | Family:Sphingomonadaceae | anaerobic degradation of aromatics |  |
|  |  | Polaromonas naphthalenivorans CJ2 | Class:Betaproteobacteria | Family:Comamonadaceae | anaerobic degradation of aromatics |  |
|  |  | Desulfovibrio desulfuricans G20 | Class:Deltaproteobacteria | Family:Desulfovibrionaceae | anaerobic degradation of aromatics;electron transfer |  |
|  |  | Azoarcus sp.EbN1 | Class:Betaproteobacteria | Family:Zoogloeaceae | anaerobic degradation of aromatics |  |
|  |  | Rhodococcus sp.RHA1 | Phylum:Actinobacteria | Family:Nocardiaceae | PAH degradation |  |
|  |  | Sphingomonas sp.AC3,Sphingomonas sp.A4 | Class:Alphaproteobacteria | Family:Sphingomonadaceae | ring-hydroxylating dioxygenase,large  subunit of PAH-dioxygenase |  |
|  |  | Mycobacterium vanbaalenii PYR-1 | Phylum:Actinobacteria | Family:Mycobacteriaceae | ring-hydroxylating dioxygenase,large  subunit of PAH-dioxygenase |  |
| Yu et al.2017 | NR | Geobacter | Class:Deltaproteobacteria | Family:Geobacteraceae | electrogenic | Microbacteriaceae,Epilithonimonas,  flexibacter |
|  |  | Proteobacteria,WS6,Firmicutes,BVA59 |  |  | electrogenic |  |
|  |  | Proteiniphilum | Class: Bacteroidetes |  | electrogenic |  |
|  |  | Thermincola | Phylum:Firmicutes;Class:Clostridia | Order:Clostridiales;  Family:Peptococcaceae | electrogenic |  |
|  |  | Proteiniphilum | Phylum:Bacteroidetes;Class:Bacteroidetes | Order:Bacteroidales | PAH degradation |  |
|  |  | Pseudoxanthomonas | Class:Gammaproteobacteria | Family:Xanthomonadaceae | PAH degradation |  |
|  |  | Methylacidiphilum | Phylum:Verrucomicrobia;  Class:Methylacidiphilae | Order:Methylacidiphilales |  |  |
|  |  | Thermomonas | Class:Gammaproteobacteria | Order:Xanthomonadales;  Family:Xanthomonadaceae |  |  |
|  |  | Bryobacter | Phylum:Acidobacteria | Order:Bryobacterales;  Family:Bryobacteraceae |  |  |
|  |  | Nevskiaceae | Class:Gammaproteobacteria | Order:Nevskiales |  |  |

Biochar amendment: CB, chicken manure; SB, wheat straw; WB, wood sawdust. GF, graphite felt; GR, graphene; CNT, carbon nanotube. nZVI, nano zero valent iron; NR, not reported.
